# Toward internal threshold for toxicological concern: in vitro hepatocyte metabolism testing of 207 chemicals using cryopreserved suspended hepatocytes for physiologically based pharmacokinetic modeling

**DOI:** 10.1093/toxsci/kfag086

**Published:** 2026-08-03

**Authors:** Corie A Ellison, Anne Marie Api, Richard A Becker, Sherry Black, Kaushal Joshi, Dan Selechnik

**Affiliations:** The Procter and Gamble Company, Mason, OH 45040, United States; Research Institute for Fragrance Materials, Woodcliff Lake, NJ 07677, United States; American Chemistry Council, Washington, DC 20002, United States; Discovery Sciences, RTI International, Durham, NC 27709, United States; Research Institute for Fragrance Materials, Woodcliff Lake, NJ 07677, United States; Research Institute for Fragrance Materials, Woodcliff Lake, NJ 07677, United States

**Keywords:** metabolism, intrinsic clearance, biotransformation, threshold of toxicological concern, PBPK modeling

## Abstract

In vitro hepatic metabolic clearance data were generated for a diverse set of 207 chemicals to advance the collaborative initiative to establish an internal Threshold of Toxicological Concern (iTTC). The data reported herein are being used for chemical-specific physiologically based pharmacokinetic (PBPK) modeling to convert oral No Observable Adverse Effect Levels (NOAELs) into estimates of internal exposure. Hepatocyte assays were conducted at 2 concentrations (0.1 and 1 µM) using cryopreserved cells from multiple species, ensuring applicability to existing mammalian toxicity studies. The metabolic clearance measurements across chemicals varied significantly, ranging from 0 to 4,294 µl/min/10^6^ cells at 0.1 µM and from 0 to 2,351 µl/min/10^6^ cells at 1 µM. A substantial proportion of the chemicals (68% at 0.1 µM and 62% at 1 µM) exhibited clearance values below 30 µl/min/10^6^ cells. Additionally, we observed a strong correlation (*R* = 0.8) between intrinsic clearance (CL_int_) values determined at the 2 concentrations. These data contribute to establishing robust iTTC values that can be utilized for: (i) extrapolating from an oral in vivo study to dermal and inhalation exposures, (ii) risk-based screening of aggregate exposures of a given substance from multiple routes of exposures, and (iii) risk-based screening of human biomonitoring results.

Metabolism is a critical biochemical process that significantly influences the pharmacokinetics of chemical substances, thereby affecting the internal exposure experienced by an organism. The rate and extent of a chemical’s metabolism directly impacts its bioavailability, duration of action, clearance from the body, and overall toxicity, making metabolism an important consideration in risk assessment and regulatory decision-making.

A collaborative effort among multiple stakeholders is underway to develop a Threshold of Toxicological Concern (TTC) based on plasma concentration, referred to as internal TTC (iTTC) (Ellison et al. 2019, 2021). As explained by Ellison et al. (2019, 2021), physiologically based pharmacokinetic (PBPK) modeling is used to translate chemical-specific oral No Observable Adverse Effect Levels (NOAELs) from the TTC database into estimates of internal exposure. To facilitate this process, high-quality in vitro metabolism data are needed for the PBPK modeling of these chemicals.

The current study aims to generate empirical in vitro hepatic metabolism clearance data for a diverse set of chemicals selected as part of the iTTC research project. These data will support the establishment of a comprehensive iTTC database, which will be vital for deriving iTTC thresholds that can be applied in exposure-based safety assessments. The test chemicals represent a subset of compounds included in existing toxicological threshold databases (Munro et al. 1996; Yang et al. 2017; Patel et al. 2020) and were chosen based on criteria outlined in Ellison et al. (2021), ensuring relevance and applicability to contemporary risk assessment frameworks.

In vitro metabolic assays were performed using hepatocytes that correspond to the species utilized in mammalian toxicity studies within the TTC database. This methodological alignment enhances the relevance of the findings and promotes the integration of the data into existing frameworks for toxicity assessment. By advancing the understanding of the in vitro metabolism of these chemicals, this study contributes to the overarching goal of establishing robust iTTC values, thereby improving safety evaluations and regulatory practices.

## Materials and methods

The chemicals listed in Table S1 of File S1 were tested in a single laboratory using a standardized protocol (File S2) for conducting in vitro hepatocyte clearance studies, with details on supplier, lot number, purity, and storage conditions provided for each. Phenacetin and verapamil HCl served as reference chemicals and their information is detailed in Table S2 of File S1.

Working stock solutions (100x the target test/reference chemical incubation concentration) were prepared in media by dilution of secondary stock solutions. For accurate addition of test chemicals that were incubated in individual vials per time point (100 μl), working stocks were prepared in dimethyl sulfoxide (DMSO) and added (1 μl) so that final DMSO concentration was 1%.

A suitable analytical method was developed for each test and reference chemical. Techniques included liquid chromatography (LC)–tandem mass spectrometry (MS/MS) and gas chromatography (GC)-MS/MS. The analytical technique used for each test chemical is shown in Table S1 of File S1 and analytical method details for each chemical are provided in Files S2–S4.

Pooled cryopreserved hepatocytes from young adult Sprague–Dawley rats, CD-1 mice, New Zealand White rabbits and/or Beagle dogs, each pool from at least 3 donors, were obtained from commercial suppliers. All incubations were carried out with 1:1 pools of hepatocytes from male and female animals. The suppliers and lot numbers of each batch of hepatocytes used are shown in Table S1 of File S2.

The metabolic activity of each lot of hepatocytes was determined using probe substrates for Phase I and Phase II enzymatic activity (File S2). Phase I activity (ethoxycoumarin O-deethylase, ECOD) was assessed by summation of 7-hydroxycoumarin (7-HC), 7-hydroxycoumarin glucuronide (7 HCG), and 7-hydroxycoumarin sulfate (7-HCS) formed. Phase II activity was assessed by formation of the 7-HCG or 7-HCS metabolite, respectively, as markers for uridine 5′-diphospho-glucuronosyltransferase (UGT) and sulfotransferase (SULT) activity.

For each species, preliminary studies were conducted to generate data on reference chemical clearance under the proposed test conditions. An assay of each reference chemical (1 μM) was conducted over 3 d for each species to assess inter-day reproducibility.

In most cases, a standard assay condition was used for incubations of test and reference chemicals with hepatocytes. Test chemicals (0.1 and 1.0 μM) and reference chemicals (1.0 μM) were incubated in triplicate wells with cryopreserved suspended hepatocytes (HEP). Hepatocytes (0.5 × 10^6^ cells/ml) were added to 24-well or 48-well polystyrene cell culture plates and pre-incubated for 10 min at 37°C in a humidified incubator filled with 5% CO_2_ while mixing on a shaker at 100 rpm. Following pre-incubation, test (or reference) chemical working solutions were added to respective wells to yield final target concentrations and the plate was gently swirled to mix. The total incubation volume was 500 μl (48-well) or 1 ml (24-well) for each replicate. The final concentration of organic solvent (e.g., DMSO) in the incubation was 0.5% for the standard assay. The plate(s) were incubated under the conditions described above. At time points (default: 0, 10, 20, 40, 60, and 120 min; a shortened time course of 0, 3, 6, 10, 15, and 20 min was used for a few chemicals) following addition of the test (or reference) chemical, the plate was removed from the shaker, placed on a 37°C heating mat, and moved in a star pattern to gently mix, then a 50-μl aliquot of the incubation mixture was removed and added to an appropriate volume of cold quench solvent in a 96-well plate or 0.75 ml matrix tubes, capped, and vortexed. The quenched reaction mixtures were vortexed and stored at ≤ −70°C prior to analysis.

Alternate incubation protocols were used for some test chemicals. Silylated glass incubation vessels (arranged in 24-well plate format) were used for chemicals that either demonstrated poor recovery from plastic plates during preliminary testing or their Log *K*_ow_ >5. For those incubations, the total volume was 1 ml and all other incubation parameters were the same as above. Volatile chemicals were incubated in individual closed glass vials per time point/replicate. In this protocol, the incubation volume was 100 μl and test chemicals were added in 100 or 10 μM solution to a final DMSO concentration of 1%. Otherwise, all incubation procedures were the same as described above for the standard assay.

Negative controls for the assay include test chemicals incubated with heat-inactivated hepatocytes (HIHEP) and without biological material (media only, MEDIA). HIHEP controls were performed in duplicate, whereas MEDIA controls were run in singlet. Negative control incubations mirrored HEP incubations, using HIHEP or MEDIA instead of HEP. Samples were collected and processed at the same time points as HEP incubations.

For each incubation concentration tested, the peak area/IS response was converted to a percent remaining based on the mean time zero peak area/IS response as 100. The percent remaining versus time data was plotted on a semilog plot (3 replicates per time point). The slope of the log_10_ (percent remaining) versus time data was calculated, with data from all 3 replicates included in the calculation of a single slope. The time course that yielded the best coefficient of determination (r^2^) was used in estimation of clearance. An *F*-test (α = 0.10) was used to determine whether the slope is significantly different from zero and this is indicated in the results (Table S4 of File S1). First-order elimination rate constants (*k*; 1/min) were calculated from negative slopes as: *K* = −2.3 × slope. In vitro intrinsic clearance rates (CL_int_; µl/min/10^6^ hepatocytes) were calculated by dividing estimated *k* values by the concentration of hepatocytes (10^6^ hepatocytes/ml).


$${\mathrm{CL}}_{\mathrm{int}}=\;\frac{k}{\mathrm{hepatocyte}\;\mathrm{concentration}}\;\;\times \;\;1,000\;µl/\mathrm{ml}$$

Several quality control measures were implemented into different aspects of this work and are summarized in File S2.

## Results/discussion

The results of the metabolic capacity testing for each species are shown in Table S4 of File S2, along with the activities reported by the hepatocyte supplier. The measured activities are generally in very good agreement (i.e., within 30%) with the reported values. Exceptions were SULT activity in dog hepatocytes (64% of the reported value), ECOD activity in mouse (55% of the average male/female reported value), and UGT activity in rat (ca. 50% or 150% of the reported values, indicating high variability in UGT measurements). Because there was no trend indicating decreased activity across multiple enzymes, the hepatocyte lots were judged to be acceptable for use.

The results of the reference chemical clearance trials are summarized in Table S5 of File S2. As expected, clearance of the reference chemicals varied by species. Average phenacetin clearance was similar for rat and mouse (19.02 and 17.77 μl/min/10^6^ cells), but higher in dog (25.05 μl/min/10^6^) and rabbit (57.18 μl/min/10^6^). Clearance of verapamil is similar in dog and mouse (23.56 and 25.47 μl/min/10^6^, respectively), but ∼2-fold higher in rat and rabbit (45.53 and 48.90 μl/min/10^6^).

**Fig. 1.**
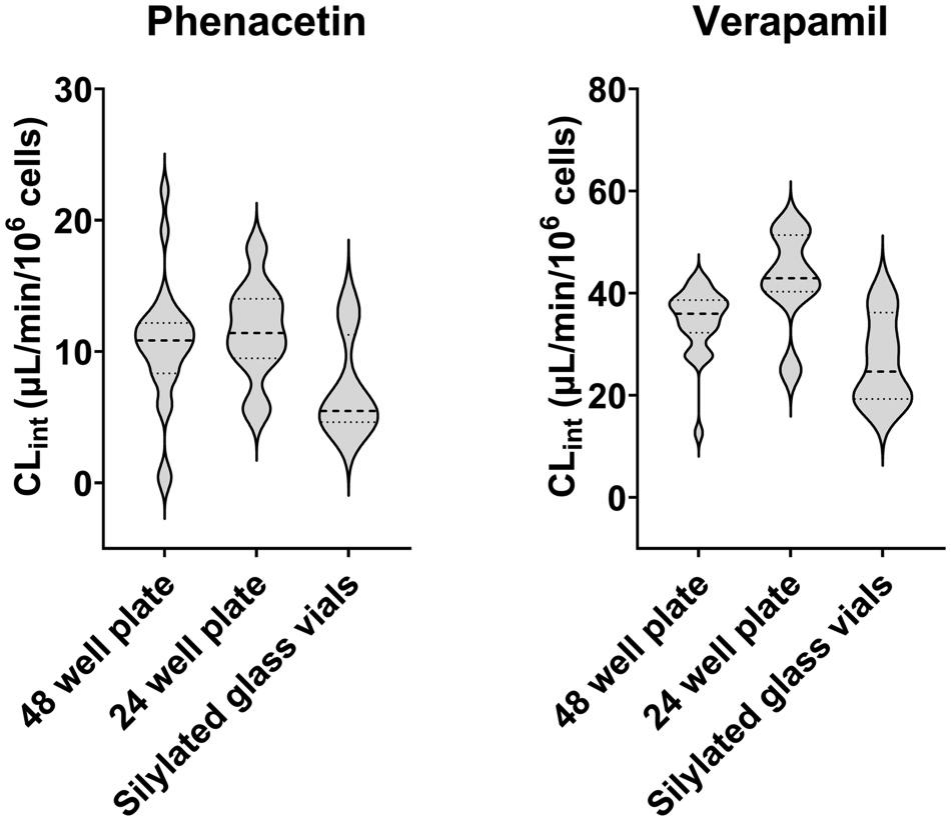
Clearance rates of reference chemicals in rat hepatocytes by assay format. The dark dashed line indicates the median, and the lighter dashed lines represent the quartiles.

The reference chemicals were included with each assay of test chemicals and the reference chemical clearance data are presented in Table S3 of File S1. For dog and rabbit, the reference chemical clearance rates were similar to those determined during preliminary studies (Table S5 of File S2). For mouse, the reference chemical CL_int_ measured during the definitive assays was 1.6- to 2-fold higher than those determined in preliminary studies. No clear reason for that discrepancy was found. A total of 49 assays were conducted with rat hepatocytes. The assays for verapamil and phenacetin performed reasonably well in the 3 assay formats used (Fig. 1). The average phenacetin CL_int_ was 10.65 ± 4.26 µl/min/10^6^ cells, which was about 57% of the average value from the preliminary studies. The verapamil CL_int_ was 37.28 ± 9.12 µl/min/10^6^ cells, which was about 76% of the average value from the preliminary studies. Variability (% CV) of phenacetin CL_int_ was about the same across the definitive runs (40.0%) as in the preliminary (36.8%). Variability of verapamil CL_int_ was lower across the definitive runs than for the preliminary assays (24.5% vs. 31.9%). The reference chemical results for all runs were deemed acceptable.

**Fig. 2.**
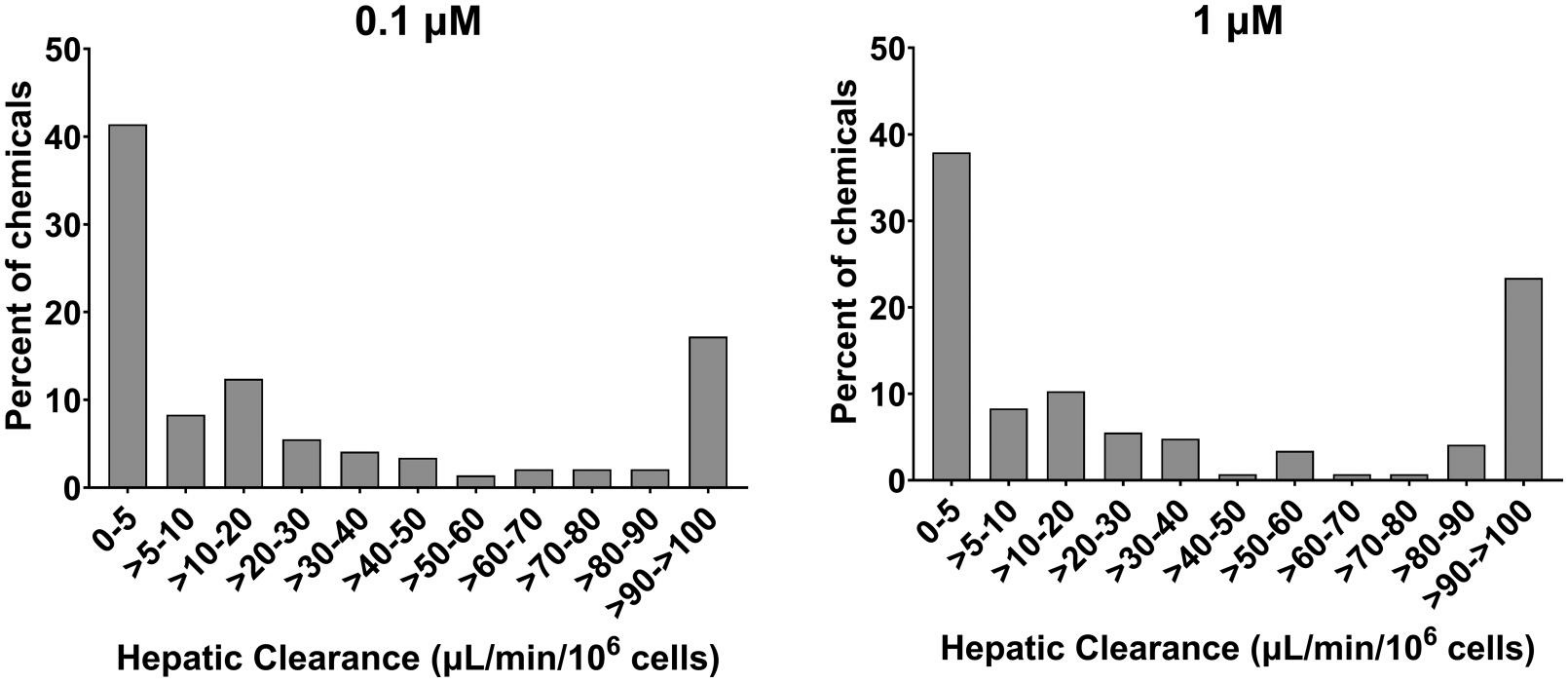
Distributions of the in vitro hepatic clearance measurements at 0.1 (left) and 1 μM (right) substrate concentrations.

The clearance results for each test chemical are summarized in Table S4 of File S1. The metabolic clearance measurements ranged from 0 to 4,294 and 0 to 2,351 μl/min/10^6^ cells at the 0.1 and 1 μM concentrations, respectively (Fig. 2). The distribution of metabolic clearance measurements was similar between the 0.1 and 1 μM concentrations. Most (68% for 0.1 μM and 62% for 1 μM) of the chemicals had clearance values < 30 μl/min/10^6^ cells.

Assays conducted on 21 chemicals failed to yield data suitable for clearance determination at either test concentration. Table S6 of File S2 shows the affected test chemicals and suspected reasons for the assay failures.

A total of 96 test chemical/concentration datasets had active cell depletion curves where slopes were not significantly different from zero and 13 test chemical/concentration datasets had nonsensical nonzero positive slopes. Clearance rates for these datasets, while obviously low, should be considered indeterminant. Clearance rates calculated for the vast majority (87 of the 96) of the low clearance test chemical/concentration datasets were <5 μl/min/10^6^ cells. For 9 (of the 96) of the low clearance datasets, variability in the data led to the failed *F*-test, but HEP clearance values, which should still be considered indeterminant, ranged up to 69.3 μl/min/10^6^ cells. The hepatocyte suspension assay used in this study has limited sensitivity for compounds with low intrinsic clearance, representing one of its primary methodological limitations. Although more advanced in vitro systems, such as hepatocyte co-cultures and 3D spheroid models, can offer improved sensitivity and sustained metabolic activity (Kukla et al. 2024; Umehara et al. 2025), inclusion of these approaches was beyond the scope of the present work.

Some chemicals had at least one test concentration where the depletion curve contained 3 or fewer time points. Anilazine, clofentezine, haloxyfop methyl, dimethyl phthalate, patulin, 2-methyl-4-phenylpentanol, cinnamyl alcohol, alpha-amyl cinnamaldehyde, 4-hexylresorcinol, and allyl isovalerate were all re-run with a shortened time course after apparent rapid clearance was observed in the standard assay. This resulted in more time points with measurable test chemical for determination of the slope of the depletion curve.

The inactive cell and media controls can be used to detect compounds with nonspecific losses due to volatility, poor stability, or other processes. The impact that nonspecific losses have on the determination of active cell CL_int_ determination should be considered. In cases of high nonspecific loss in the inactive cells, the active cell CL_int_ values may be overestimated. Volatility is a likely cause where CL_int_ is similar for active cells and both controls as demonstrated by 1,1,1,2-tetrachloroethane, alpha-methylstyrene, p-dichlorobenzene, tetrachloroethylene, and trichloroethylene. Poor solubility in aqueous solution also can lead to nonspecific losses; generally, limited solubility can become a concern for compounds with Log *K*_ow_ > 5. For these chemicals, the apparent loss in cell-free (MEDIA) wells is typically greater than in the inactive cell controls, which may be due to their poor aqueous solubility. However, loss in the inactive controls is generally low for these chemicals, perhaps due to improved solubility in the presence of the lipids and proteins from the inactivated cells, when compared with the active cell depletion rate. Other possible nonspecific loss processes include chemical instability and adsorption.

**Fig. 3.**
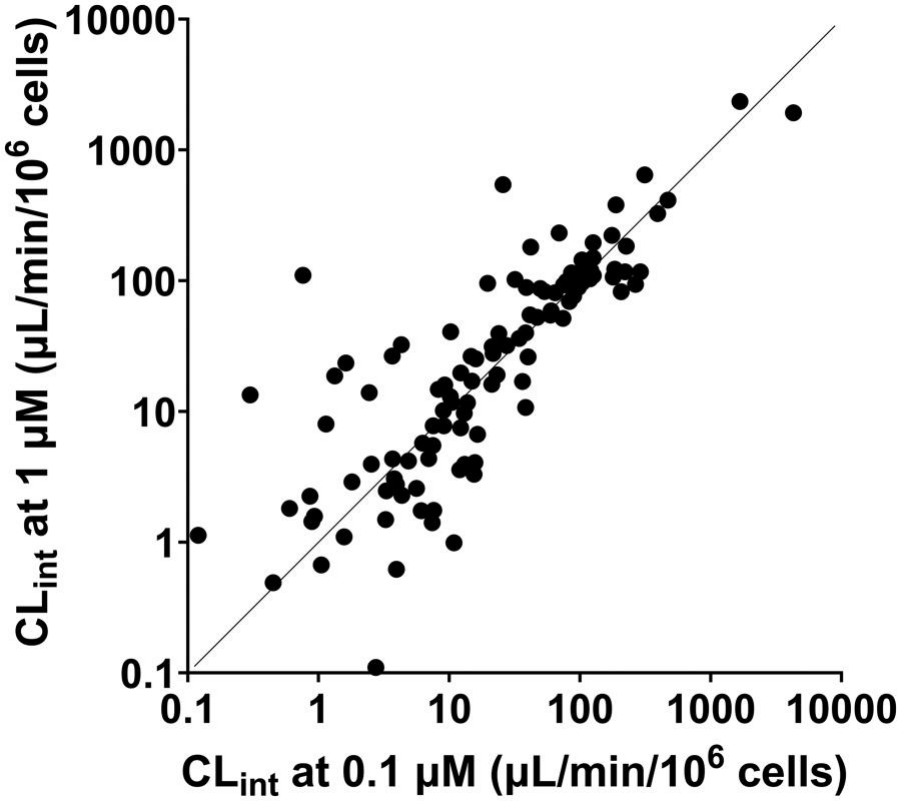
Correlation of in vitro hepatic clearance measurements at 0.1 and 1 μM substrate concentrations. Solid line represents the line of unity.

**Fig. 4.**
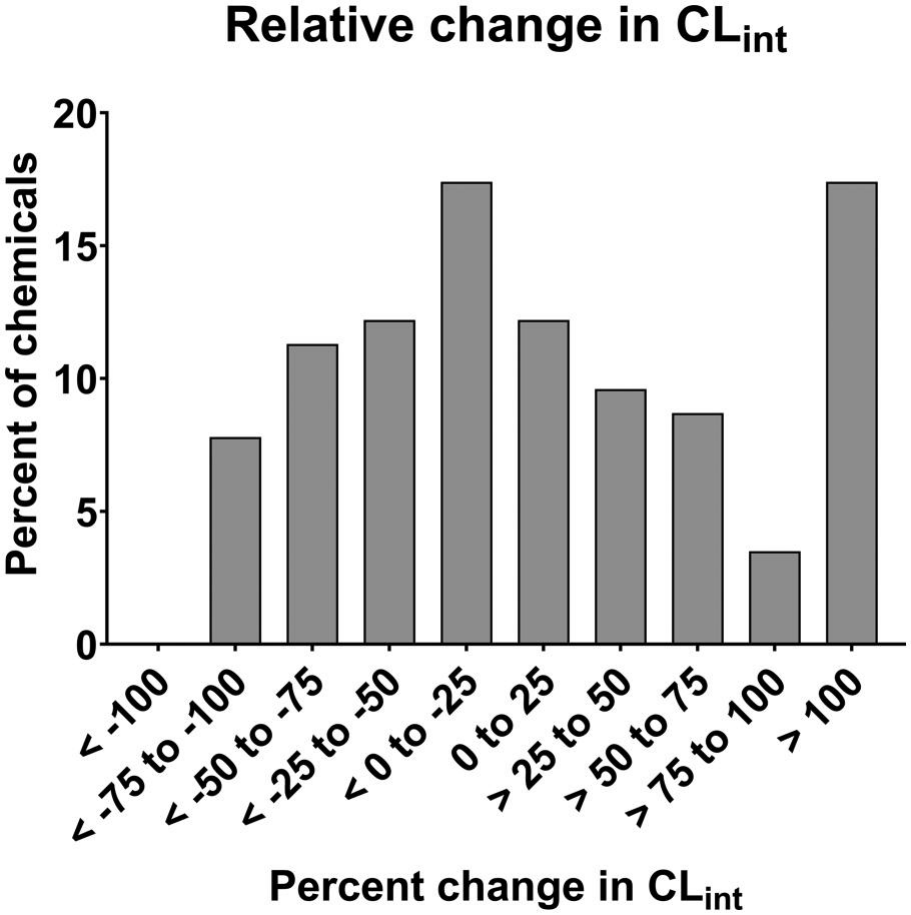
Relative percent change in CL_int_ determined for test chemicals at the 0.1 and 1 μM substrate concentrations. Percent change calculated as: ((CL_int_ 1 μM − CL_int_ 0.1 μM)/CL_int_ 0.1 μM) × 100.

The correlation and concentration-dependence of CL_int_ was assessed for all chemicals yielding a depletion rate at both the 0.1 and 1 μM test concentrations. Negative values were set to zero because they reflect lack of or little-to-no metabolism. A total of 145 chemicals fulfilled this criterion. The correlation (*R*) was 0.8 between CL_int_ determined at 0.1 and 1 μM (Fig. 3). The chemical-specific CL_int_ value determined at 0.1 and 1 μM test concentrations was used to assess saturation of metabolism at substrate concentrations not exceeding 1 μM. A decrease in CL_int_ with increasing test chemical concentration is indicative of potential saturation. Chemicals with CL_int_ values of zero for the 0.1 μM test concentration were excluded from the analysis to avoid divisions by zero. A total of 115 chemicals were included in the analysis of concentration-dependence (Fig. 4). The majority (81%) of chemicals had a relative difference in CL_int_ that was greater than −50%, indicating minimal saturation at the 1 μM test concentration. This analysis should be interpreted within the appropriate context, recognizing that relying solely on 2 test chemical concentrations makes it challenging to accurately assess the presence and extent of metabolic saturation.

While OECD test guidelines exist for determining in vitro intrinsic clearance using fish models (OECD TG 319A and OECD TG 319B), no such guidelines are currently established for mammalian in vitro biotransformation assays. However, an OECD guideline for mammalian models is reportedly under development, and the protocol and data presented here may contribute to that effort. In the interim, researchers have developed best practices and principles for in vitro biotransformation assays, including efforts to systematically characterize in vitro methods for hepatic metabolic clearance and to harmonize test methods for in vitro hepatic clearance studies (Gouliarmou et al. 2018; Louisse et al. 2020).

The present data, particularly concerning reproducibility and variability observed with reference chemicals, may be valuable to others investigating similar aspects of in vitro assays. During this project, practical heuristics were developed to guide incubation conditions, potentially aiding future research. For instance, to address potential volatility and solubility concerns without preliminary testing, the following guidance was applied: (i) if Henry’s law constant > 10 Pa·m³/mole, individual vials were used per time point to reduce volatility loss; (ii) if Log *K*_ow_ > 5, test chemicals were added in DMSO solution to minimize solubility concerns; (iii) if both conditions were met, individual vials and DMSO addition were employed; and (iv) otherwise, standard incubation conditions were used.

Furthermore, this dataset may be beneficial for other applications, such as evaluating in vitro mass balance models (Armitage et al. 2014, 2021; Scherer et al. 2026) or improving workflows that integrate biotransformation data with such models. While some data points may exhibit uncertainty or variability (e.g., those with very low or high CL_int_ values), the ability to categorize a chemical’s clearance rate (e.g., negligible, low, moderate, high) can still be sufficiently useful depending on the decision context.

In conclusion, this study generated in vitro hepatocyte clearance data for a diverse set of chemicals, providing valuable insights for future research, human safety assessments, and regulatory decisions. These results will serve as a foundational resource for projects focused on developing internal thresholds of toxicological concern and other approaches/models related to metabolism and pharmacokinetics. Once the PBPK modeling is complete and a distribution of internal exposures exists, the results can be applied to support a number of different risk-based decision contexts, including extrapolating from an oral in vivo study to dermal and inhalation exposures, and screening of aggregate exposures of a given substance from multiple sources and routes of exposures. In addition, in the absence of a chemical-specific internal or external reference dose, iTTC could be used as a health protective Biomonitoring Equivalent for interpreting human biomonitoring data in a risk context (Becker et al. 2012). If the steady-state plasma concentration of such a substance detected in a human biomonitoring study is found to be less than the iTTC, then it could be presumed that, at that internal exposure level, this substance would present *de minimis* risk to human health.

## Supplementary Material

kfag086_Supplementary_Data

## References

[kfag086-B1] Armitage JM , Sangion A, Parmar R, Looky AB, Arnot JA. 2021. Update and evaluation of a high-throughput in vitro mass balance distribution model: IV-MBM EQP v2.0. Toxics. 9:315. 10.3390/toxics9110315 34822706 PMC8625852

[kfag086-B2] Armitage JM , Wania F, Arnot JA. 2014. Application of mass balance models and the chemical activity concept to facilitate the use of in vitro toxicity data for risk assessment. Environ Sci Technol. 48:9770–9779. 10.1021/es501955g 25014875

[kfag086-B3] Becker RA , Hays SM, Robison S, Aylward LL. 2012. Development of screening tools for the interpretation of chemical biomonitoring data. J Toxicol. 2012:941082. 10.1155/2012/941082 22518117 PMC3306934

[kfag086-B4] Ellison CA , Api AM, Becker RA, Efremenko AY, Gadhia S, Hack CE, Hewitt NJ, Varcin M, Schepky A. 2021. Internal threshold of toxicological concern (iTTC): where we are today and what is possible in the near future. Front Toxicol. 2:621541. 10.3389/ftox.2020.621541 35296119 PMC8915896

[kfag086-B5] Ellison CA , Blackburn KL, Carmichael PL, Clewell HJ III, Cronin MTD, Desprez B, Escher SE, Ferguson SS, Grégoire S, Hewitt NJ, et al 2019. Challenges in working towards an internal threshold of toxicological concern (iTTC) for use in the safety assessment of cosmetics: discussions from the cosmetics Europe iTTC Working Group Workshop. Regul Toxicol Pharmacol. 103:63–72. 10.1016/j.yrtph.2019.01.016 30653989 PMC6644721

[kfag086-B6] Gouliarmou V , Lostia AM, Coecke S, Bernasconi C, Bessems J, Dorne JL, Ferguson S, Testai E, Remy UG, Brian Houston J, et al 2018. Establishing a systematic framework to characterise in vitro methods for human hepatic metabolic clearance. Toxicol In Vitro. 53:233–244. 10.1016/j.tiv.2018.08.004 30099088 PMC10288526

[kfag086-B7] Kukla DA , Belair DG, Stresser DM. 2024. Evaluation and optimization of a microcavity plate-based human hepatocyte spheroid model for predicting clearance of slowly metabolized drug candidates. Drug Metab Dispos. 52:797–812. 10.1124/dmd.124.001653 38777596

[kfag086-B8] Louisse J , Alewijn M, Peijnenburg AACM, Cnubben NHP, Heringa MB, Coecke S, Punt A. 2020. Towards harmonization of test methods for in vitro hepatic clearance studies. Toxicol In Vitro. 63:104722. 10.1016/j.tiv.2019.104722 31756541

[kfag086-B9] Munro IC , Ford RA, Kennepohl E, Sprenger JG. 1996. Correlation of structural class with no-observed-effect levels: a proposal for establishing a threshold of concern. Food Chem Toxicol. 34:829–867. 10.1016/s0278-6915(96)00049-x 8972878

[kfag086-B10] Patel A , Joshi K, Rose J, Laufersweiler M, Felter SP, Api AM. 2020. Bolstering the existing database supporting the non-cancer threshold of toxicological concern values with toxicity data on fragrance-related materials. Regul Toxicol Pharmacol. 116:104718. 10.1016/j.yrtph.2020.104718 32603678

[kfag086-B11] Scherer MN , Feshuk M, Armitage JM, Arnot JA, Crizer D, DeVito M, Ferguson SS, Freeman K, Honda GS, Harrill JA, et al 2026. Characterizing accuracy of model predictions for chemical concentration in high throughput screening assays. Environ Sci Technol. 60:4869–4877. 10.1021/acs.est.5c10195 41627972

[kfag086-B12] Umehara K , Klammers F, Walter I, Naik T, Dudal V, Remus T, Ekiciler A, Anderka I, Pugliano A, Preiss L, et al 2025. Prediction of hepatic metabolic clearance in rats and dogs using long-term cocultured hepatocytes. Drug Metab Dispos. 53:100055. 10.1016/j.dmd.2025.100055 40203525

[kfag086-B13] Yang C , Barlow SM, Muldoon Jacobs KL, Vitcheva V, Boobis AR, Felter SP, Arvidson KB, Keller D, Cronin MTD, Enoch S, et al 2017. Thresholds of toxicological concern for cosmetics-related substances: new database, thresholds, and enrichment of chemical space. Food Chem Toxicol. 109:170–193. 10.1016/j.fct.2017.08.043 28867342

