# Supplementary material for: Toward internal threshold for toxicological concern: in vitro hepatocyte metabolism testing of 207 chemicals using cryopreserved suspended hepatocytes for physiologically based pharmacokinetic modeling
Source: Toxicol Sci. 2026 Aug 3;209(8):kfag086. doi: 10.1093/toxsci/kfag086 (PMC13476076; doi:10.1093/toxsci/kfag086)
Supplement: Supplementary file 1 [file kfag086_supplementary_data.zip › Supplemental File 2.docx]

**Supplemental files**

Supplemental file 1 – Excel document. Summary of test chemicals, reference chemicals, and the results from in vitro hepatocyte incubations.

Supplemental file 2 – Word document. Additional information related to methods and results.

Supplemental file 3 – Excel document. Analytical methods for hepatocyte incubations tagged as “HEP #-ACC”

Supplemental file 4 – Excel document. Analytical methods for hepatocyte incubations tagged as “HEP #-RIFM”

**Chemicals:**

**Test Chemicals:**

The chemicals shown in Table 1 of Supplemental file 1 were tested in in vitro hepatocyte clearance assays. The supplier, lot number, purity, and storage conditions are presented for each.

**Reference chemicals:**

Phenacetin and verapamil were selected as reference chemicals. The supplier, lot number, purity, and storage conditions for the reference chemicals are presented in Table 2 of Supplemental File 1.

**Solution Preparation:**

**Test and Reference Chemical Stock Solutions:**

A solution (10 mM) of each test and reference chemical was prepared in water, or if insoluble in

water, in dimethyl sulfoxide (DMSO). Dronabinol (Δ9-THC) was received as a solution in methanol.

Nalidixic acid, Na salt was prepared in acetonitrile. Secondary stock solutions (1 mM) were prepared by dilution of the primary solution to 1 mM in the same solvent used for the primary stock. All primary and

secondary stock solutions were stored at ≤ -70 °C and used within six months of preparation.

**Test and Reference Chemical Working Solutions:**

Working stock solutions (100x the target test/reference chemical incubation concentration) were

prepared in media by dilution of secondary stock solutions. For accurate addition of test chemicals that

were incubated in individual vials per time point (100 μL), working stocks were prepared in DMSO and

added (1 μL) so that final DMSO concentration was 1%.

**Stopping Solutions:**

Ice cold acetonitrile, methanol, ethyl acetate or 0.1% formic acid in acetonitrile, dependent on the analytical method requirements was used as the stopping solution.

**Internal Standard Solutions:**

LC-MS/MS internal standard (IS) stock solutions were prepared in DMSO at 1 mg/mL and then were

diluted to 1–2 μg/mL in acetonitrile or acetonitrile containing 0.1% formic acid. Most GC/MS internal

standard stock solutions were prepared in DMSO at 1 mM and diluted to 0.5–1 μM in ethyl acetate.

Some GC/MS IS stocks were prepared at 1 mg/mL DMSO and then were diluted in ethyl acetate to the

final concentration. IS solutions were stored at -20 °C.

**Analytical Methods:**

A suitable analytical method was developed for each test and reference chemical. Techniques included liquid chromatography (LC)–tandem mass spectrometry (MS/MS) and gas chromatography (GC)-MS/MS. For determination of in vitro metabolic clearance, quantitation was by peak area response. Appropriate internal standards were used to monitor for sample processing error. The analytical technique used for each test chemical is shown in Table 1 of Supplemental File 1 and analytical method details for each chemical are provided below and in Supplemental files 3 and 4.

**LC/MS System 1**

This system consisted of an API 4000TM Triple Quadrupole Mass Spectrometer with Turboion Spray source with an Agilent 1000 LC system. This system was operated in positive or negative ion mode depending on the analyte. The default chromatography column was a Waters Atlantis dC18 (50 x 3 mm) with C18 guard cartridge, though in some cases other columns were used. For positive ion methods, the mobile phases were typically either (1) A: 10 mM aqueous ammonium formate with 0.1% formic acid and B: methanol containing 10 mM ammonium formate and 0.1% formic acid, or (2) A: 0.1% aqueous acetic acid and B: methanol containing 0.1% acetic acid. For negative ion methods, the mobile phases were typically A: 0.1% aqueous acetic acid and B: 0.1% acetic acid in methanol. Mobile phase gradients typically began with 90–98% A and, after a short hold (0.5 min) changed to 95% B.

**LC/MS System 2**

This system consisted of a SCIEX Qtrap 6500+ Mass Spectrometer with an ExionLCTM system. This system was operated in positive or negative ion mode depending on the analyte. Chromatography columns used included an Acquity UPLC BEH C18 (1.7 μm, 2.1 x 100 mm), an Atlantis T3 C18 (5 μm, 2.1 x 100 mm), an Acquity UPLC HSS T3 (1.8 μm, 2.1 x 100 mm) or a Phenomenex Kinetex PFP (1.7 μm, 2.1 x 100 mm). For positive ion methods, the mobile phases were typically A: 5- or 10-mM aqueous ammonium formate with 0.1% formic acid and B: methanol containing 5- or 10-mM ammonium formate and 0.1% formic acid. For negative ion methods, the mobile phases were typically either (1) A: 0.1% aqueous formic acid and B: 0.1% formic acid in methanol, or (2) A: 5 mM ammonium acetate with 0.1% acetic acid and B: 0.1% acetic acid in methanol. Mobile phase gradients typically began with 90–98% A and, after a short hold (0.5 min) changed to 90–98% B.

**GC/MS System 1**

This system consisted of an Agilent Technologies 7000 Triple Quadrupole Mass Spectrometer with EI source with an Agilent 7890A GC system and an Agilent GC Sampler 80. Separations were achieved using either an Agilent J&W DB-5MS (30 m, 0.25 mm ID, 0.25 μm film thickness) or an Agilent J&W DB-624 (30 m, 0.32 mm ID, 1.8 μm film thickness) chromatography column.

**GC/MS System 2**

This system consisted of an Agilent Technologies 5977 Single Quadrupole Mass Spectrometer with EI source with an Agilent 7890B GC system and an Agilent GC Sampler G4514A. Separations were achieved using an Agilent J&W DB-624 (30 m, 0.32 mm ID, 1.8 μm film thickness) chromatography column.

**GC/MS System 3**

This system consisted of an Agilent Technologies 5973 Single Quadrupole Mass Spectrometer with EI source with an Agilent 6890 GC system and a Leap CombiPAD Autosampler. Separations were achieved using an Agilent J&W DB-5MS (30 m, 0.25 mm ID, 0.25 μm film thickness).

**Hepatocyte Procedures**

Pooled cryopreserved hepatocytes from young adult Sprague Dawley rats, CD-1 mice, New Zealand White rabbits and/or Beagle dogs, each pool from at least 3 donors, were obtained from commercial suppliers. Cryopreserved hepatocytes from mouse and rabbit were obtained as single sex lots and were combined upon thawing to create a 1:1 pool. All incubations were carried out with 1:1 pools of hepatocytes from male and female animals. The cryopreserved hepatocytes were stored in a liquid nitrogen vapor phase dewar until use. The suppliers and lot numbers of each batch of hepatocytes used is shown in Table 1.

**Table 1. Hepatocyte Source and Lot Numbers**

| **Species** | **Strain** | **Lot #** | **Sex** | **Supplier** |
| --- | --- | --- | --- | --- |
| Dog | Beagle | QZP | Mix | BIOIVT |
| Mouse | ICR/CD-1 | HEJ | Male | BIOIVT |
|  |  | YSC | Female | BIOIVT |
| Rabbit | New Zealand White | TDV | Male | BIOIVT |
|  |  | LFG | Female | BIOIVT |
| Rat | Sprague-Dawley | XVJ | Mix | BIOIVT |

Hepatocytes were removed from cryogenic storage and placed in a 37 °C water bath to thaw. Once thawed, the cells were immediately poured into a 50-mL conical tube containing 44-mL of prewarmed INVITROGRO HT thawing media, each cryotube was rinsed with thawing media (1.5 mL) and rinsate was added to the conical tube. The hepatocytes were resuspended by gently inverting the tube several times and then centrifuged at 50g for 5 min at room temperature. The supernatant was removed by aspiration without disturbing the cell pellet, and then the cell pellet was resuspended with approximately 2 mL of room temperature INVITROGRO KHB media per thawed cryotube used by gently swirling. A 50-μL aliquot of the homogenous cell suspension was transferred to a counting tube containing Trypan Blue. The concentration and viability were determined by Trypan Blue exclusion using a hemocytometer. Additional INVITROGRO KHB media was added to the cell suspension to achieve a cell density of 0.5 x 10^6^ cells/mL.

Heat inactivated hepatocytes (HIHEP) were prepared from the hepatocytes that were thawed and resuspended to 0.5 x 10^6^ cells/mL, as described above, and inactivating them at 85 °C in a water bath for 15 min. The HIHEP mixtures prepared in advance of their use were stored at -20 °C until needed.

**Confirmation of Phase I and Phase II Metabolic Activity**

The metabolic activity of each lot of hepatocytes was determined using probe substrates for Phase I and Phase II enzymatic activity Phase I activity (ethoxycoumarin O-deethylase, ECOD) was assessed by summation of 7-hydroxycoumarin (7-HC), 7-hydroxycoumarin glucuronide (7 HCG), and 7-hydroxycoumarin sulfate (7-HCS) formed. Phase II activity was assessed by formation of the 7-HCG or 7-HCS metabolite, respectively as markers for uridine 5'-diphospho-glucuronosyltransferase (UGT) and sulfotransferase (SULT) activity.

**Table 2. Conditions for Metabolic Activity Testing of Hepatocytes**

| **Hepatocyte Concentration** | **Probe Substrate and Concentration (µM)** | **Incubation Time (min)** | **Enzyme** |
| --- | --- | --- | --- |
| 1 x 10^6^ cells /mL | 7-ethoxycoumarin (75) | 60 | Phase I (CYP) |
|  | 7-hydroxycoumarin (30) | 60 | Phase II (Glucuronidation and Sulfation) |

The incubations for enzyme activity were performed on one day per species in triplicate wells in 48-well polystyrene plates using INVITROGRO KHB media. Hepatocytes (0.5 x 106 cells/mL) were thawed and resuspended. Hepatocytes were pre-incubated for ~10 min at 37 C in a humidified incubator filled with 5% CO2 while mixing on a shaker at about 100 rpm. Following pre-incubations, substrate solution was added to respective wells to yield final target concentrations and mixed gently by swirling the plate. The total incubation volume was 500 μL for each replicate. The final concentration of organic solvent (DMSO) in incubation was ≤0.5. The plate(s) were incubated and at 60 min following addition of substrate, the plate was removed from the shaker, placed on a 37 C heating mat and an aliquot (50 μL) was removed after gently mixing the reaction mixture by pipetting up/down and added to prefilled 150 μL cold methanol in 0.75-mL matrix tubes in 96-well plate format. The quenched reaction mixtures were vortexed and stored at ≤ -70 C prior to analysis.

**Standards**

Stock solutions were prepared in MeOH at 1 mM (7-HCG and 7-HCS) and 6 mM 7 HC. These solutions were serially diluted to prepare working stocks ranging from 100 to 20,000 nM (7 HCG), 10 to 10,000 nM (7-HCS), and 10 to 2,000 nM for 7-HC. At least 6 points were used to prepare each curve. Working stocks (50 μL) were diluted in cold methanol (150 μL) in 0.75-mL matrix tubes in 96 well plate format, vortexed for 5 min at 1200 rpm, and centrifuged at 4000 rpm (~3724g) for 5 min. Supernatant (10 μL) was transferred to a 96-well plate along with 90 μL of 50:50 H2O: MeOH, and 5 μL of 2,4-dichlorophenoxyacetic acid (2,4-D) internal standard solution (IS, 2000 ng/mL in acetonitrile). The plate was vortexed for 5 min at 800 RPM prior to analysis by LC/MS using the method described below.

**Sample Analysis**

The quenched reaction mixtures were thawed and vortexed for 5 min at 1200 rpm, and centrifuged at 4000 rpm (~3724g) for 5 min. Then 10 μL of supernatant was transferred to a 96-well plate, diluted with methanol, IS added and mixed. The chromatographic separation was achieved using a Phenomenex Luna C18 (150 x 2 mm, 4 μm, 100A) column with C18 guard cartridge. The mobile phases were A: 10 mM aqueous ammonium formate with 0.1 formic acid and B: methanol containing 10 mM ammonium formate and 0.1 formic acid. The mobile phase gradient began with 85 A and, after a 1 min hold, changed to 98 B over 4 min and held for 2 min before returning to initial conditions. The column temp was 25 C and flow rate was 0.5 mL/min. The mass spectrometer was operated in negative ion mode with CAD 8, CUR 16, GS1 45, GD2 55, IS -3500, TEM 650, EP 10, and dwell 75 msec. Analyte specific mass spectrometry conditions are given in the table below.

**Table 3. Mass Spectrometry Parameters for Metabolic Capacity Assay Analytes**

| **Analyte** | **Q1** | **Q3** | **DP** | **DE** | **CXP** |
| --- | --- | --- | --- | --- | --- |
| 2,4-D (IS) | 218.8 | 160.5 | -45 | -16 | -11 |
| 7-HC | 160.8 | 132.6 | -55 | -50 | -19 |
| 7-HCS | 240.8 | 160.6 | -45 | -22 | -11 |
| 7-HCG | 337 | 160.5 | -55 | -28 | -11 |

**Activity Calculation**

ECOD activity (pmol/min/106 cells) was calculated by summation of 7-HC, 7 HCG, and 7-HCS formed with normalization to incubation time and hepatocyte concentration. UGT and SULT activity was calculated by normalizing the amount of 7-HCG or 7-HCS formed, respectively, to time and hepatocyte concentration.

**Table 4. Phase I and Phase II Enzymatic Activities**

| **Species** | **Lot #** | **Sex** | **Data Source** | **Phase 1 (ECOD)^a^** | | **Phase 2 (UGT)^a^** | | **Phase 2 (SULT)^a^** | |
| --- | --- | --- | --- | --- | --- | --- | --- | --- | --- |
|  |  |  |  | **Mean** | **SD** | **Mean** | **SD** | **Mean** | **SD** |
| Dog | QZP | Pooled | Measured | 389.7 | 20.5 | 181.2 | 11.7 | 119.1 | 3.2 |
|  | QZP | Pooled | COA^b^ | 299 | - | 240 | - | 185 | - |
| Mouse | - | Pooled | Measured | 116 | 3.7 | 262 | 2.1 | 92.9 | 5.4 |
|  | HEJ | Male | COA^b^ | 189 | - | 283 | - | 52 | - |
|  | YSC | Female | COA^b^ | 232 | - | 274 | - | 165 | - |
| Rabbit | - | Pooled | Measured | 525.7 | 81.7 | 354.4 | 8.4 | 184.3 | 6.9 |
|  | TDV | Male | COA^b^ | 590 | - | 218 | - | 281 | - |
|  | LFG | Female | COA^b^ | 669 | - | 349 | - | 105 | - |
| Rat | XVJ | Pooled | Measured | 112.8 | 13.1 | 435.6 | 49.1 | 91.6 | 34.2 |
|  | XVJ | Pooled | COA^b^ | 105 |  | 276 |  | 86.3 |  |
| ^a^ All activities expressed as pmol/min/10^6^ cells. | | | |  |  |  |  |  |  |
| ^b^ These are activities reported by the hepatocyte supplier on the Certificates of Analysis. | | | | | | |  |  |  |

**Preliminary Studies – Reference Chemicals**

For each species, preliminary studies were conducted to generate data on reference chemical

clearance under the proposed test conditions. Assay of each reference chemical (1 μM) was conducted

on three days for each species.

**Table 5. Reference Chemical Clearance Trial Results**

| **Species** | **Inc ID** | **Test Chemical** | **Test Conc (µM)** | **ClInt** | **Mean** | **SD** | **RSD** |
| --- | --- | --- | --- | --- | --- | --- | --- |
|  |  |  |  | **(µL/min/10^6^ cells)** | **(µL/min/10^6^ cells)** |  |  |
| Rat | Hep#1 | Phenacetin | 1 | 25.8 | 19.02 | 7 | 36.8 |
| Rat | Hep#5_11_5_19 | Phenacetin | 1 | 11.82 |  |  |  |
| Rat | Hep#6_11_5_19 | Phenacetin | 1 | 19.45 |  |  |  |
| Mouse | Hep#2_10_17_19 | Phenacetin | 1 | 25.29 | 17.77 | 6.87 | 38.65 |
| Mouse | Hep#9_11_7_19 | Phenacetin | 1 | 11.81 |  |  |  |
| Mouse | Hep#10_11_13_19 | Phenacetin | 1 | 16.22 |  |  |  |
| Rabbit | Hep#3_10_18_19 | Phenacetin | 1 | 55.42 | 57.18 | 3.44 | 6.02 |
| Rabbit | Hep#11_11_14_19 | Phenacetin | 1 | 61.15 |  |  |  |
| Rabbit | Hep#12_11_14_19 | Phenacetin | 1 | 54.98 |  |  |  |
| Dog | Hep#2_10_17_19 | Phenacetin | 1 | 30.29 | 25.05 | 4.63 | 18.48 |
| Dog | Hep#7_11_6_19 | Phenacetin | 1 | 21.54 |  |  |  |
| Dog | Hep#8_11_6_19 | Phenacetin | 1 | 23.31 |  |  |  |
| Rat | Hep#1 | Verapamil | 1 | 60.27 | 45.53 | 14.51 | 31.86 |
| Rat | Hep#5_11_5_19 | Verapamil | 1 | 31.26 |  |  |  |
| Rat | Hep#6_11_5_19 | Verapamil | 1 | 45.07 |  |  |  |
| Mouse | Hep#2_10_17_19 | Verapamil | 1 | 29.48 | 25.47 | 4.1 | 16.09 |
| Mouse | Hep#9_11_7_19 | Verapamil | 1 | 25.64 |  |  |  |
| Mouse | Hep#10_11_13_19 | Verapamil | 1 | 21.29 |  |  |  |
| Rabbit | Hep#3_10_18_19 | Verapamil | 1 | 40.65 | 48.9 | 8.04 | 16.45 |
| Rabbit | Hep#11_11_14_19 | Verapamil | 1 | 49.33 |  |  |  |
| Rabbit | Hep#12_11_14_19 | Verapamil | 1 | 56.72 |  |  |  |
| Dog | Hep#2_10_17_19 | Verapamil | 1 | 24.12 | 23.56 | 3 | 12.75 |
| Dog | Hep#7_11_6_19 | Verapamil | 1 | 26.24 |  |  |  |
| Dog | Hep#8_11_6_19 | Verapamil | 1 | 20.32 |  |  |  |

**Incubations of Test and Reference Chemicals with HEP**

In most cases, a standard assay condition was used for incubations of test and reference chemicals with hepatocytes. Test chemicals (two concentrations, default: 0.1 μM and 1.0 μM) and reference chemicals (one concentration, 1.0 μM) were incubated in triplicate wells with cryopreserved hepatocytes (HEP). Hepatocytes (0.5 x 10^6^ cells/mL) were added to 24-well or 48-well polystyrene cell culture plates and pre-incubated for 10 min at 37 °C in a humidified incubator filled with 5% CO2 while mixing on a shaker at 100 rpm. Following pre-incubation, test (or reference) chemical working solutions were added to respective wells to yield final target concentrations and the plate gently swirled to mix. The total incubation volume was 500 μL (48 well) or 1 mL (24 well) for each replicate. The final concentration of organic solvent (e.g., DMSO) in the incubation was 0.5% for the standard assay. The plate(s) were incubated under conditions described above. At time points (default: 0, 10, 20, 40, 60, and 120 min; a shortened time course of 0, 3, 6, 10, 15 and 20 min was used for a few chemicals) following addition of test (or reference) chemical, the plate was removed from the shaker, placed on a 37 °C heating mat and moved in a star pattern to gently mix, then a 50-μL aliquot of the incubation mixture was removed and added to an appropriate volume of cold quench solvent in a 96-well plate or 0.75 mL matrix tubes, capped and vortexed. For LC/MS analysis sample volumes (50 or 100 μL) were quenched with 3 volumes of solvent. For GC/MS analysis, samples (100 μL) were quenched with 100 μL of solvent. The quenched reaction mixtures were vortexed and stored at ≤ -70 °C prior to analysis.

Alternate incubation protocols were used for some test chemicals. Glass incubation vessels

(silylated glass 15–45 mm 1-dram vials (Fisher #0333930C) arranged in 24 well plate format) were used

for chemicals that either demonstrated poor recovery from plastic plates during preliminary testing or their LogP >5. For those incubations, the total volume was 1 mL and all other incubation parameters were the same as above. Volatile chemicals were incubated in individual closed glass vials (Analytical Sales and Services #96F01) per time point/replicate. In this protocol, the incubation volume was 100 μL and test chemicals were added in 100 or 10 μM solution to a final DMSO concentration of 1%. Otherwise, all incubation procedures were the same as described above for the standard assay.

Negative controls for the assay include test chemicals (at the same concentrations indicated above) incubated in the presence of heat-inactivated hepatocytes (HIHEP) and in the absence of any biological material (media only, MEDIA). HIHEP controls were carried out in duplicate while MEDIA controls were run in singlet. MEDIA controls for reference chemicals were included for every assay. The reference chemical test concentration in negative controls was 1 μM. Negative control incubations were conducted similarly to HEP incubations, substituting HIHEP or MEDIA for HEP. Samples were collected at the same time points as for HEP and processed and stored as for the HEP incubations.

**Calculations**

For each incubation concentration tested, the peak area/IS response was converted to a percent remaining based on the mean time zero peak area/IS response as 100. The percent remaining versus time data was plotted on a semilog plot (three replicates per time point). The slope of the log (percent remaining) versus time data was calculated, with data from all three replicates included in the calculation of a single slope. The time course that yielded the best coefficient of determination (r2) was used in estimation of clearance. An F-test (α=0.10) was used to determine whether the slope is significantly different from zero and this is indicated in the results (Table 4 of Supplemental File 1). If the test chemical peak disappears in live cell incubations before the third time point, the depletion curve was considered too limited (as it would only contain 2 datapoints) to report a clearance rate. Intrinsic clearance was estimated based on the elimination rate constant and normalized to hepatocyte cell number in the incubation. Calculations were as follows:

CL = V_max_/K_m_ or 0.693/t_1/2_ (half-life); assumes [S] << Km

Half-life: (t_1/2_)(min) = 0.693⁄−𝑘; where k is the terminal elimination rate constant.

Slope *2.3 = -k

Intrinsic clearance (CL_int_) (μL/min/10^6^ hepatocytes) = 𝑘⁄10^6^ ℎ𝑒patocytes/𝜇L

**Quality Control**

An analytical method to detect each test chemical in incubation media was developed, and linearity over a known range, typically 0.01–1 μM was demonstrated using appropriate standards. Dilutions of test chemical spiking solutions in media were analyzed and peak area compared with peak area of corresponding time zero incubation samples to confirm accuracy of incubation spiking and identify unexpected or rapid chemical loss. Analytical results were reviewed and tabulated, and data links were reviewed by technical staff for accuracy.

A standardized spreadsheet was used for data tabulation/calculations. The spreadsheet contains details of the in vitro assay conditions which were reviewed for accuracy. The analytical results are included in the spreadsheet and the tabulated data were electronically linked to summary data tabulation sheets. Plotted depletion data were examined visually to select the initial linear depletion range. Assay peak areas were compared with peak area of low standards and samples with peak areas less than the low standard peak area were excluded from the depletion curve. The spreadsheet displays the number of time points included in the depletion curves.

Incubation results were summarized on the summary tab in each individual incubation Excel file and then linked electronically to the Final Summary spreadsheet. The incubation Excel filename was included in the Final Summary spreadsheet to allow trackback to the original data file.

In addition, concurrent controls were used to monitor changes in test chemical concentration in the presence of inactive cells or cell media only which could indicate non-enzymatic chemical losses as described in the results. Also reference chemicals were run on each incubation day, using the same cell preparation as was used for the test chemical assays and day-to-day performance was monitored.

**Failed Assays**

Assays conducted on 21 chemicals failed to yield data suitable for clearance determination at either test concentration. The table below shows the affected test chemicals and suspected reasons for the assay failures.

**Table 6. Failed Assays**

| **Suspected Cause of Assay Failure** | **Test Chemicals** |
| --- | --- |
| Endogenous or Other Analytical Interference | Sucrose, Phenylalanine, Leucine, Tryptophan, Nicotinamide, Ethanolamine, 1-Amino-2-propanol, p-Anisidine HCl, 2,4-Dichlorobenzyl alcohol, Styrene |
| Poor Stability | Hydralazine, tert-Butyl peroxybenzoate |
| Low or Variable Analytical Response | Methyl viologen hydrate (hydrate of parquat dichloride), Anilazine |
| Rapid Loss in Assay | Diethyl sebacate, Disulfiram, Allyl heptanoate, 2-pentylfuran, diallyl phthalate |
| Analytical Failure | Cumene, Trimethylamine HCl |

**Standardized Protocol**

**PROTOCOL TITLE**: **Developing In Vitro Metabolic Clearance Data to Support Development of the Internal Threshold of Toxicological Concern**

# **3.0** **Materials and Equipment**

## **3.1 Test Chemicals**

ACC will choose approximately 180 chemicals for testing in the hepatocyte clearance (MC) assay and will specify species for testing. If no species is specified, the default species will be rat. RTI will obtain each chemical with high reported purity, with purity of >98% preferred and the minimum acceptable purity of 90%. Certificates of Analysis will be maintained in study records.

## **3.2 Reference Chemicals**

Reference chemicals, with known clearance rates, will be included on each day test chemicals are assayed. Reference chemicals are intended to serve as a check that the assay is functioning properly. The two reference chemicals, phenacetin and verapamil, will each be run at a single (1 µM) concentration.

## **3.3 Equipment and Labware**

### **3.3.1 Fixed Equipment**

- Analytical balance for mg quantities - Mettler Toledo- Ultra-microbalance (UMX2)
- Vortex mixer, VWR-vortex-Genie 2
- Biological Safety Cabinet: 1300 Series A2- Class II, Type A2
- Sample incubation equipment. Common options include:
- Thermo Scientific- Model 3307 and 3310 Series CO_2_ incubator-controlled RH with Sterilization Cycle.
- VWR Microplate Shaker
  - Fisher Scientific -Slide Warmer
  - Refrigerated centrifuge, Allegra X-12 R and X-15R centrifuge
  - Small benchtop refrigerated centrifuge for microfuge tubes, Eppendorf Microcentrifuge (5415)
  - pH meter, Mettler Toledo Seven Multi
  - Compound microscope capable of 20 × magnification: Nikon Eclipse TS100 microscope
  - Vacuum pump – optional: Bel-Art hi flow vacuum aspirator collection system
  - 4 °C refrigerator
  - -20 °C freezer
  - -70 °C Ultra Cold freezer
  - Cryogenic container or dewar containing liquid nitrogen, LN2 capacity-MVE High Efficiency 800 series vapor storage temperature at -150 °C.
  - Dubnoff - metabolic shaking water bath incubator with temperature control.
  - Eppendorf - MixMate Microplate Shaker
  - Branson Ultrasonic Sonicator
  - PlateLoc Thermal Microplate Sealer

### **3.3.2 Labware**

- Glassware for preparing and storing chemical solutions
- Rainin- Single and Multichannel Pipette 10, 200, 300, 1000 µL and their tips
- Adjustable tip spacing pipettes - transfer of multiple samples between labware of different sizes and formats
- Serological pipets 1, 2, 5, 10, and 25 mL individually wrapped sterile non pyrogenic.
- Portable Pipet-aid
- Corning Costar Flat Bottom Cell Culture Plates (48 well, Corning™ 3548)
- Cluster tubes (1.2 mL, polypropylene, Corning 4408)
- Matrix™ 0.75/1.4mL round bottom tubes (ThermoFisher 4170 and 4140)
- Pierceable TPE caps for capping 96 individual tubes Micronic
- 1.5 mL microcentrifuge tubes and their racks
- Disposable Vacuum Filter/Storage systems (Corning™ 430767)
- Timer, Traceable four-Channel Alarm Timer (S407992)
- Disposable hemacytometers (Neubauer improved), Incyto Co. C-Chip, DHC-N01-5, VWR (cat. no. 82030-468 USA)
- Cryogloves to remove cells from liquid nitrogen
- 50 mL tube rack
- 50 mL conical centrifuge tubes, Thermo Scientific™ Nunc™ EZFlip™ Conical Centrifuge Tubes (50 mL) and rack
- Spatulas for weighing chemicals

## **3.4 Other Chemicals and Cell Culture Reagents**

### **3.4.1 Internal standards and solvents**

- Internal standards (TBD)
- Stopping and extraction solvents, HPLC grade (e.g. acetonitrile, methylene chloride)
- Solvents to dissolve test chemicals, HPLC grade (e.g. DMSO, acetone, acetonitrile)

### **3.4.2 Reagents and Media**

- Sodium hydroxide (NaOH, 1 M) for pH adjustment
- Hydrochloric acid (HCl, 1 M) for pH adjustment
- Hepatocyte thawing medium (e.g., InVitroGRO HT medium)
- Hepatocyte incubation medium (e.g., InVitroGRO HI medium)
- Trypan blue, Gibco ™, 0.4%

## **3.5 Biological Material**

Pooled cryopreserved hepatocytes from young adult Sprague Dawley rats, CD-1 mice, New Zealand White rabbits and/or Beagle dogs, each pool from at least 3 donors. Cryopreserved hepatocytes from male and female of a species may be obtained as separate preparations with the same cell concentration and combined 1:1 upon thawing. All incubations must be carried out with 1:1 mixtures of hepatocytes from male and female animals. The cryopreserved hepatocytes will be obtained from a commercial vendor in compliance with any and all federal regulations and stored in a liquid nitrogen vapor phase dewar until use. All cryopreserved hepatocytes will be characterized for metabolic competence by the vendor and the vendor will supply a datasheet of these characterization results. All hepatocyte lots will also be evaluated in-house for metabolic capacity – see Section 5.3.

Heat-inactivated (HI) hepatocytes (HIHEP) for each species noted above will be prepared as described below in Section 5.2.

# **4.0 Preparation of Solutions**

## **4.1 Test and Reference Chemical Stock Solutions**

A solution (10 mM) of each test and reference chemical will be prepared in water, or if insoluble in water, in DMSO. The stock solution will be labeled with (at least) chemical name, concentration, solvent, storage conditions and preparation date. Secondary stock solutions (1 mM) will be prepared by dilution of the primary solution to 1 mM in the same solvent used for the primary stock. All primary and secondary stock solution preparation information will be recorded on appropriate worksheets; test chemical weights recorded to at least three significant figures. All primary and secondary stock solutions will be stored at ≤ ‑70 °C, and used within one month of preparation.

## **4.2 Test and Reference Chemical Spiking Stock Solutions**

Spiking stock solutions (100 x the target test/reference chemical incubation concentration) will be prepared by dilution of primary and secondary stock solutions in the same solvent. Record spiking stock preparation information on appropriate worksheets.

## **4.3 Stopping Solutions**

Default test chemical stopping solution will be ice cold acetonitrile. If necessary, to meet requirements of the analytical method for particular test chemicals, alternative solvents may be used. Record stopping solution used for each assay on assay worksheets.

## **4.4 Internal Standards Solutions**

Appropriate internal standards will be selected for each test chemical during analytical method development. Internal standard solution preparation details will be recorded on appropriate worksheets.

# **5.0 Procedures**

## **5.1 Thawing of Cryopreserved Hepatocytes and Suspension in Incubation Media**

1. Prewarm hepatocyte thawing medium (e.g., INVITROGRO HT Medium) to 37 °C.

NOTE: Instructions are given for thawing three cryovials. No more than 3 cryovials may be combined in a single 50 mL of thawing medium. If fewer vials are thawed together, volumes of thawing medium used must be adjusted so the final total volume (including cell suspension) is 50 mL.

2. Transfer 44 mL to a sterile 50-mL conical tube.
3. Immerse the cryovial(s) containing HEP in a 37 °C water bath, taking care to keep the cap above the water line. Keep the vial(s) in the water bath until thawing is just complete (generally 1-2 minutes); some small ice crystals should still be visible within the vial.
4. Empty the contents of the hepatocyte vial into the tube containing the pre-warmed hepatocyte thawing medium.
5. Rinse each cryotube with 1.5 mL of pre-warmed hepatocyte thawing medium and decant or pipet the contents into the conical tube.
6. Resuspend the hepatocytes by gently inverting the tube 3 times.
7. Centrifuge at 50 x g at room temperature for 5 min.
8. Discard the supernatant by either decanting in a single motion or aspirating using a vacuum pump.
9. Add approximately 0.8 mL of hepatocyte incubation medium for every 1 x10^6^ cells and invert the tube gently to resuspend the hepatocytes.
10. Determine the viability and cell count of the pooled hepatocytes via Trypan blue exclusion with a hemacytometer. The cell count is performed in duplicate.
11. Adjust the concentration of hepatocytes to 0.5 x 10^6^ viable cells/mL in hepatocyte incubation medium.
12. Perform 2 additional cell counts after final dilution of cells to confirm concentration. NOTE: We will determine in preliminary experiments whether we can accurately count cells at this concentration. If that is not possible, cell concentrations will be based on the count determined in step 10 and the appropriate dilution factor.
13. Keep the cell suspension on ice until use.
14. The hepatocytes will not be used if the cell viability is < 70%.

## **5.2 Preparation of Heat Inactivated Hepatocytes (HIHEP)**

Suspensions of HEP in hepatocyte incubation medium, prepared as described above (~0.5 x 10^6^ cells/mL), will be placed in a boiling water bath for 15 minutes. The volume of the suspension after boiling will be adjusted to the initial (pre-boiling) volume by addition of hepatocyte incubation medium INVITROGRO HI. The HIHEP mixture will be aliquoted into tubes and stored at -20 °C until needed.

## **5.3 Confirmation of Phase I/II Metabolic Activity for HEP**

**Note: These assays will be conducted for each lot of hepatocytes.**

The metabolic activity of the HEP will be determined using probe substrates for Phase I and Phase II enzymatic activity (Table 1). Phase I activity (ECOD) is assessed by summation of 7-HC, 7‑HCG, and 7-HCS formed. Phase II activity is assessed by formation of the 7HCG or 7-HCS metabolite, respectively for UGT and SULF. The incubations for enzyme activity will be performed on one day (per lot of hepatocytes) in triplicate wells in 48-well polystyrene plates. Measured metabolic activity should be within ±30% of the values given on the hepatocyte COA. If the metabolic activity is not within range on the first day of testing, two additional independently thawed tubes of hepatocytes will be assayed and the mean activities will be determined. Any cell lots that do not yield metabolic activity within ±30% of the values given on the hepatocyte COA will not be used.

Table 1. Conditions for Metabolic Activity Testing of Hepatocytes

| **Hepatocyte Concentration** | **Probe Substrate and Concentration (µM)** | **Incubation Time (min)** | **Enzyme** |
| --- | --- | --- | --- |
| 1 x 10^6^ cells /mL | 7-ethoxycoumarin (75) | 60 | Phase I (CYP) |
|  | 7-hydroxycoumarin (30) | 60 | Phase II (Glucuronidation and Sulfation) |

## **5.4 Preliminary Studies – Reference Chemicals**

For each species, preliminary studies will be conducted to generate data on reference chemical clearance under the proposed test conditions. Assay of each reference chemical will be conducted on three days for each species. Incubation conditions will be as described in Section 5.5. Data generated will be compared with species specific literature data, where available, and will serve as historical control data for reference chemical clearance. Literature data for some species and reference chemicals are presented in the Appendix.

## **5.5 Preliminary Studies – Solubility Testing**

In conjunction with analytical method development, test chemicals (in organic solvent) will be spiked (to final expected test concentrations) into hepatocyte media in a 48-well culture plate and glass vials and incubated at 37 °C for 2 h. An aliquot (50 µL) will be removed from each (after mixing) and processed as in Section 5.6 for incubation samples. Recovery of test article will be assessed. This recovery data, along with available physical chemical property data (e.g., Kow, water solubility), will be used to help predict whether plastic or glass incubation vessels will allow best recovery.

## **5.6** **Incubations of Test and Reference Chemicals with HEP**

**Note: Default incubation conditions are described here. It may be necessary to adjust test chemical concentrations, hepatocyte concentration, time points and/or incubation vessels for some compounds. Any modifications to the default conditions will be documented on assay worksheets and noted in the report.**

Test chemicals (two concentrations, default: 0.1 µM and 1.0 µM) and reference chemicals (one concentration, 1.0 µM) will be incubated in triplicate wells with cryopreserved hepatocytes (HEP, suspended in media as described above). Hepatocytes (0.5 x 10^6^ cells/mL) will be added to 48-well polystyrene cell culture plates and pre-incubated for ~10 minutes at 37 °C in a humidified incubator filled with 5% CO2 while mixing on a shaker at about 100 rpm. Following pre-incubation, test (or reference) chemical working solutions will be added to respective wells to yield final target concentrations and the plate gently swirled to mix. The total incubation volume will be 500 µL for each replicate. The final concentration of organic solvent (e.g., DMSO) in incubation will be ≤0.5% whenever possible, with the highest allowable solvent concentration of 1%. If solvent concentrations above 0.5% are used this must be noted and reference chemical data will be evaluated to determine any effects. The plate(s) will be incubated under conditions described above. At time points (0, 10, 20, 40, 60, and 120 min) following addition of test (or reference) chemical, the plate will be removed from the shaker, placed on a 37 °C heating mat and moved in a star pattern to gently mix, then a 50-µL aliquot of the incubation mixture will be removed and added to an appropriate volume of cold quench solvent in a 96-well plate or 0.75 mL matrix tubes, capped and vortexed. The default quench solvent is 150 µL cold acetonitrile, but an alternate may be required depending on the analytical requirements for each test chemical. Sampling and/or quench volumes may vary according to analytical method requirements and will be documented on worksheets. The quenched reaction mixtures will be vortexed and stored at ≤ -70 °C prior to analysis.

For some test chemicals it may be necessary to use glass incubation vessels based on results of preliminary aqueous media recovery testing. Those assays will be conducted in sylilated glass 15-45 mm 1-dram vials (Fisher #0333930C) arranged in 24 well plate format. Otherwise all incubation procedures will be the same as described above. Any other modifications to the incubation procedure (i.e., test chemical concentrations, hepatocyte concentration or time points) will be documented and reported.

## **5.7** **Negative Control Incubations**

Negative controls for the assay include test chemicals (at the same concentrations as used in Section 5.4) incubated in the presence of heat-inactivated hepatocytes (HIHEP) and in the absence of any biological material (media only, MEDIA). HIHEP controls will be carried out in duplicate while MEDIA controls will be run in singlet. For reference chemicals, HIHEP controls will be included for the first three assays (as applicable) for each species. If the data show abiotic loss of ≤15% for reference chemicals, the reference chemical HIHEP controls may be discontinued for subsequent assays. MEDIA controls for reference chemicals will be included for every assay. The reference chemical test concentration in negative controls will match that used in Section 5.4.

Negative control incubations will be conducted similarly to HEP incubations (Section 5.5), substituting HIHEP or media for HEP. Samples will be collected at the same time points as for HEP and processed and stored as for the HEP incubations.

## **5.8 Spiking Stock Samples**

Duplicate dilutions of spiking stocks (5 µL) will be prepared in quench solvent (495 µL) in glass screw capped vials, vortexed and stored under the same conditions as incubation samples until analysis. For analysis, an aliquot will be diluted with incubation buffer and additional quench solvent to prepare a sample with the same solvent:buffer ratio as the incubation samples. For example, under default assay sampling conditions as described above, a 50-µL aliquot of the spiking stock dilution, will be diluted with 50 µL incubation buffer and 100 µL quench solvent. These samples will be prepared for analysis identically to the incubation samples. Peak area response will be compared with the peak area response of separately prepared stocks to check that incubation concentrations were on target.

# **6.0** **Analytical Method Development**

A suitable analytical method will be developed for each test and reference chemical. Available techniques include liquid chromatography (LC)–tandem mass spectrometry (MS/MS), gas chromatography (GC)-MS/MS, and high-performance LC (HPLC) with ultraviolet (UV) or fluorescence detection. For determination of in vitro metabolic clearance, quantitation is by peak area response. Appropriate internal standards are used to monitor for sample processing error. To streamline method development activities, a generic method is used for each technique, with minor adjustments, as needed, to optimize for each test chemical. HPLC and LC-MS sample preparation usually consists of a protein precipitation with added solvent and use of internal standards. GC-MS sample preparation is typically through solid-phase extraction (SPE) techniques.

# **7.0 Sample Analysis**

Samples will be analyzed using the appropriate analytical methods. Typically, for HPLC or LC-MS/MS methods, sample plates will be thawed at room temperature. The samples will be mixed on a plate vortexer, and then centrifuged to precipitate protein. Supernatants are transferred and appropriate internal standards are added to analysis plates (or vials) and analyzed. An Analysis Stock of each test article or reference chemical will be prepared in solvent. Three suitable dilutions in incubation buffer will be prepared for analysis similarly to assay samples and will be analyzed with assay samples to demonstrate linearity over the expected test article concentration range.

## **7.1 Analysis and Calculations**

For each incubation concentration tested, the peak area/IS response will be converted to a percent remaining based on the mean time zero peak area/IS response as 100%. The percent remaining versus time data will be plotted on a semilog plot (three replicates per time point). The slope of the log (percent remaining) versus time data will be calculated, with data from all three replicates included in the calculation of a single slope. Linear regression analysis may be carried out on various time sets (i.e., possibly excluding one or more time points) to determine any differences in correlation for various time courses. The time course that yields the best coefficient of determination (r^2^) will be used in estimation of clearance. An F-test (α=0.10) will be used to determine whether the slope is significantly different from zero. Initially, chemicals with slopes not significantly different from zero will be marked as ‘not reportable’ due to the lack of a well-characterized depletion profile under the current conditions, though an alternative method of handling low clearance rates may be added in a protocol amendment. Similarly, if the test chemical peak disappears in live cell incubations before the third time point, the depletion curve will be considered too limited (as it would only contain 2 datapoints) to report a clearance rate. Follow-up experiments may be conducted with adjustments to cell concentration and/or sampling times to better characterize the clearance. If test chemical concentration in HIHEP controls is depleted more than 20% over the time course, abiotic loss mechanisms must be considered and follow-up experiments with alternate protocols (i.e., in glass and/or capped vials) may be needed.

Intrinsic clearance will be estimated based on the elimination rate constant and normalized to hepatocyte cell number in the incubation.

Calculations are as follows:

CL = V_max_/K_m_ or 0.693/t1/2 (half-life); assumes [S] << K_m_

Half-life: (t_1/2_)(min) = ${0.693}/{-k}$; where k is the terminal elimination rate constant.

Slope *2.3 = -*k*

Intrinsic clearance (CL_int_) (µL/min/10^6^ hepatocytes) = $k/{10^{6} hepatocytes/\mu L}$

# **12.0 References**

Floby, E, Breim, S, Terelius, Y and Sohlenius-Sternbeck, A-K, 2004, Use of a cocktail of probe substarates for drug-metabolizing enzymes for the assessment of the metabolic capacity of hepatocyte preparations. *Xenobiotica*, 34: 949-959.

Lu, C, Gallegos, R, Uttamsingh, V, Xia, CQ, Miwa GT, Balani, SK and Gan, L-S, 2006, Comparison of Intrinsic Clearance in Liver Microsomes and Hepatocytes from Rats and Humans: Evaluation of Free Fraction and Uptake in Hepatocytes. *Drug Metab. Dispo*., 34:1600-1605.

Wood, FL, Houston, JB and Hallifax, D, 2017, Clearance prediction methodology needs fundamental improvement: trends common to rat and human hepatocytes/microsomes and implications for experimental methodology, *Drug Metab Dispo.,* 45:1178-1188.

**A****ppendix A: Reference Chemical Clearance**

| **Reference Chemical** | **Species** | **Test System** | **Reported Cl** | **Clint (µL/min/10^6^ cells)** | **Source** |
| --- | --- | --- | --- | --- | --- |
| Phenactin | Rat | Hepatocytes | 0.58 L/h/kg | 1.59 | Lu_2008. |
| Phenacetin | Rat | Hepatocytes | 14.30 µL/min/10^6^ cells | 14.30 | Floby 2004 |
| Phenacetin | Dog | Hepatocytes | 13.70 µL/min/10^6^ cells | 13.70 | Floby 2004 |
| Verapamil | Rat | Hepatocytes | 664 mL/min/kg | 138.3 | Wood, 2012 |
